# How different are the differences? A commentary on the paper “Beat-based dancing to music has evolutionary foundations in vocal learning”

**DOI:** 10.1186/s12868-024-00850-7

**Published:** 2024-11-06

**Authors:** Virginia B. Penhune

**Affiliations:** 1 Laboratory for Motor Learning and Neural Plasticity, Department of Psychology, Concordia University, Montréal, QC Canada; 2 Montréal Laboratory for Brain, Music and Sound (BRAMS), Montréal, QC Canada

## Abstract

We propose that while examining homologies and convergences in auditory-motor synchronization between humans and non-human animals is informative, examining differences in behaviour and brain mechanisms can help to better define the boundaries of the phenomena.

There are few researchers in the domain of music neuroscience who have exerted such a strong and deeply reasoned theoretical influence on central issues in our field. For this reason, it is a pleasure to read Ani Patel’s new paper and to have the opportunity to comment on it. In this paper, Patel extends his ideas about the link between vocal learning and beat synchronization in non-human animals to the domain of dance. His previous experimental work has provided evidence that some vocal-learning birds show partial abilities to synchronize and flexibly move the beat of human music [1, 2]. In parallel, other labs have shown that non-human primates can also be trained to synchronize to a beat from visual stimuli [3]. Based on these findings, Patel has proposed that this type of partial synchronization in birds and in non-human primates may be a precursor of the broader human ability to move to music [4]. Further, he speculates that avian synchronization is based on gene-regulation changes in the dorsal auditory-motor circuits that control vocalization [5]. In the current paper, he puts forward the idea that these adaptations in the vocal-motor control system fortuitously spread to optimize control of the motor system more generally, specifically via enhanced auditory-parietal connectivity. He hypothesizes that these adaptations are present in some primates, and especially in humans, which is what allows us to synchronize a wide range of movements to complex auditory sequences such as those found in music and dance.

The search for animal-human behavioural homologies and converging brain mechanisms is a powerful source of hypotheses about possible evolutionary and neurophysiological changes that might underlie the development of specific behaviours. In the current paper, Patel sets out several testable hypotheses based on his ideas. First, he proposes that in humans, individual differences in beat synchronization ability should be related to individual differences in the white matter connections between auditory, parietal and premotor regions. Some evidence for this hypothesis comes from work showing that individual differences in the connections between auditory and premotor regions are related to the ability to synchronize speech to a stream of syllables [6]. In the domain of music, learning to reproduce rhythms, and tapping and continuation to a beat have been linked to white matter microstructure in the arcuate fasciculus [7, 8]. Second, he suggests that parietal to premotor connections should be enhanced in humans compared to primates. Information about differences in the auditory-parietal-prefrontal connections between humans and primates is available from the comparative neuroanatomical work of Michael Petrides [9] who suggests that differential connectivity with high-order frontal motor regions and enhanced development of frontal processing are key contributors to language and other cognitive development in humans [10]. In a recent study, connectivity between inferior parietal cortex and premotor regions was found to be related to second-language learning [11]. In particular, connections with more superior premotor regions were related to improvements in vocabulary, and connections with more inferior regions were related to improvements in rate of articulation. Patel also raises several other interesting directions for future research, including examining the role of parietal cortex in beat processing and developmental studies of synchronization abilities in children.

Examining homologies between humans and non-human animals has been demonstrated to be informative in many domains of cognitive neuroscience. However, differences in behaviour and brain mechanisms are likely to be equally important and have the potential to help us better define the boundaries of the phenomena to be explored. In this current adaptation of the vocal-learning hypothesis, a central tenet is that synchronization to the beat is a key point of convergence between human and animal responses to music, and in turn, auditory-motor synchronization is considered a core feature of human dance. Let us consider these points. When viewed with human eyes, Snowball the parrot makes a variety of convincingly dance-like movements in response to music [2]. This kind of synchronized movement is a highly salient feature of many forms of human dance; but it seems likely that dance and non-instrumental vocal music vastly predate most of the familiar musics that we call to mind when making this comparison. It seems potentially equally likely that regular, sound-producing movement is a precursor to music, rather than an outcome of it, a point that has been made previously by Steven Brown [12].

Further, even vocal-learning birds who exhibit partial synchronization to human music do not appear to synchronize to other stimuli in the wild, or to synchronize to music without at least passive feedback from their human partners. Macaques who can learn to produce synchronous movements do so most accurately to visual stimuli, and only after extensive, reward-based training [13]. Examples of chimpanzee “dances” and “drumming” although intriguing, are notable not just for limited synchronization, but for a lack of sequential or hierarchical organization, and limited variety and novelty of movement. Human dancers don’t just walk or swing their arms, but create elaborate, complex movements that are not part of their everyday repertoire. These examples raise interesting questions about the differences between animal and human behaviour that could be fruitful to explore along with homologies and convergences. Why, for example, do some vocal-learning birds learn to move to music with only passive feedback from humans while primates do not? Patel speculates that these birds are more intrinsically motivated than other animals to learn the structure of complex auditory sequences. If this is the case, we can ask why this motivation is related to synchronization and how this informs our understanding of auditory behaviour in other animals. Similarly, what are the differences between primates and humans that make auditory information more relevant for us than for them? How do other higher-order cognitive capacities in primates, such as auditory memory, and the ability to make hierarchical sequential predictions influence the observed differences in synchronization behaviour in comparison with humans? Finally, why is the motivation to synchronize so strong in humans and not in other animals?

The fact that these questions are unanswered is a strength not a weakness of this paper. Patel’s proposal does what strong, grounded theory always does: prompts new thinking and moves us to test new ideas.

## Data Availability

Not applicable.
